# A Droplet Digital PCR Based Approach for Identification and Quantification of Porcine and Chicken Derivatives in Beef

**DOI:** 10.3390/foods11203265

**Published:** 2022-10-19

**Authors:** Huili Xu, Xiaoyu Ma, Zihong Ye, Xiaoping Yu, Guangfu Liu, Zhengliang Wang

**Affiliations:** Zhejiang Provincial Key Laboratory of Biometrology and Inspection and Quarantine, College of Life Sciences, China Jiliang University, Hangzhou 310018, China

**Keywords:** quantification, pork, chicken, beef, adulteration, droplet digital PCR

## Abstract

Adulteration of high-value beef with lower-priced alternatives is a world-wide problem resulting in consumers’ distrust and market chaos. Therefore, effective methods for the identification and quantification of adulterated beef products are urgently needed. In this study, we developed a reliable droplet digital PCR (ddPCR) method targeting the single-copy nuclear genes for qualitative and quantitative detection of the presence of porcine and chicken derivatives in beef. A fixed constant (transfer coefficient) was introduced to directly transform the ratio of DNA copy number to the mass proportion of targeted meats. Results revealed that the linearity range of quantification for pork and chicken were both from 1% (*w/w*) to 90% (*w/w*). The limit of detection (LOD) and limit of quantification (LOQ) of the developed ddPCR method were the same for pork and chicken in beef, with LOD 0.1% (*w/w*) and LOQ 1% (*w/w*). The accuracy and applicability of the method was tested and verified using mixed samples with the known proportions and commercially available beef products. We conclude that our developed ddPCR method was accurate and reliable in identifying and quantifying porcine and chicken derivatives in beef and therefore has great potential to be applied in routine analyses and quality control of beef products.

## 1. Introduction

With the improvement of people’s living standards and the integration of Chinese and western dietetic culture, beef accounts for an increasing proportion of the meat consumption in China. Driven by high economic profits, adulteration of meat products occurs frequently in the meat processing industry and market where high-priced beef is adulterated with lower-priced meats such as pork, chicken and duck [1,2,3]. This pattern of meat adulteration can result in consumers’ distrust and market chaos or even correlate with religious belief and social stability [4]. Therefore, it is critical to develop efficient methods for the identification and quantification of adulterated beef.

Currently, there are three main methods for the detection of meat adulteration, namely traditional morphological-based, species-specific protein-based and nucleic acid-based methods [5,6,7,8]. The traditional method is mainly based on morphological characteristics, which is unsuitable for the identification of processed meats and cannot quantify the proportion of mixed meats [9]. Protein-based methods including enzyme-linked immunosorbent assay (ELISA) [10,11], mass spectrometry (MS) [12,13] and high-performance liquid chromatography (UPLC) [14,15] are effective in meat quantification, but proteins are easily degraded and/or denatured during deep processing, leading to inaccurate and unreliable detection results. Among the nucleic acid-based methods, real-time quantitative polymerase chain reaction (qPCR) is outstanding and widely accepted for identification and quantification of meat adulteration [16,17,18]. Droplet digital PCR (ddPCR) is a more recently developed method with unprecedented sensitivity and precision as well as capacity to provide an absolute measure of nucleic acid concentration without establishing a standard curve [19]. In ddPCR, sample DNA is partitioned into tens of thousands of droplets by a droplet generator and fluorescence signals are measured via end-point measurement. This technique avoids calculating copy number of DNA from intermediary cycle threshold (Ct) value that can be affected by amplification efficiency and inhibitors, thereby increasing the overall accuracy for quantifying target DNA especially at low concentrations and/or in a high background of non-target DNA [20]. Numerous studies have evidenced the applicability of ddPCR for detection and quantification of meat adulteration [21,22,23,24,25] including determination of the presence of chicken in sheep and goat meat products [21], Atlantic salmon in processed and mixed seafood products [22] and porcine-derived materials in halal food products [23].

A crucial challenge for the quantification of adulterated meat by ddPCR is transforming copy numbers of DNA to the mass proportion of meat. Owing to the fact that copies of target genes are different among animal species or even varied greatly in different tissue types of the same kind of meat, the DNA concentration of an amplified target usually could not represent the true mass proportion [26]. So far, the most quantitative study adopted a two-step procedure to transform the ratio of DNA copy numbers to the mass proportion of meat, in which the DNA copy numbers were first transformed to the DNA content and the DNA content was then converted into the meat weight [27,28,29]. Noticeably, this approach required the establishment of two calibration curves which would increase the bias of the results. Recent research developed ddPCR quantification systems through adding an internal reference or introducing a constant (multiplication factor or conversion factor) to transform the copy numbers to the fraction of target meat species [21,30,31,32]. Such methods without two conversion steps were proved to be accurate and stable in the quantification of meat adulteration. For instance, a ddPCR method was designed based on nuclear DNA sequence and integrated with an inner reference species (Turkey) for quantification of fur-bearing animal meat (fox, mink or raccoon dog) in raw and processed food [30].

In this study, we aim to develop a ddPCR system for the accurate and precise identification and quantification of porcine and chicken derivatives in beef. To directly transform the DNA copy numbers to the mass proportion of meat, a constant (transfer coefficient) was introduced as reported previously [21] and precise quantitative calculation formulae were consequently established for quantifying the content of pork and chicken in beef products, respectively. The inner-laboratory sensitivity, repeatability, reproducibility and applicability of this method was also evaluated and validated. Our results will provide an effective method that can be used for routine quality assurance and control in beef products. 

## 2. Materials and Methods

### 2.1. Samples Preparation

Fresh meat (skeleton muscle) samples of beef (*Bos taurus*), pork (*Sus scrofa*) and chicken (*Gallus gallus*) were purchased from a local market in Hangzhou, Zhejiang, China. All the samples were washed with sterilized distilled water, minced and dried in a drying oven at 80 °C for 72 h, and subsequently ground into a superfine powder in liquid nitrogen using a mortar and pestle. A total mass of 10 g of mixed pork and beef powder were prepared to known proportions, ranging from 1% to 90% pork/beef by mass, i.e., the mixtures contained 0.1 g pork/9.9 g beef (1%), 0.5 g pork/9.5 g beef (5%), 1.0 g pork/9.0 g beef (10%), 2.0 g pork/8.0 g beef (20%), 3.0 g pork/7.0 g beef (30%), 4.0 g pork/6.0 g beef (40%), 5.0 g pork/5.0 g beef (50%), 6.0 g pork/4.0 g beef (60%), 7.0 g pork/3.0 g beef (70%), 8.0 g pork/2.0 g beef (80%) and 9.0 g pork/1.0 g beef (90%), respectively. Different mass proportions of chicken and beef were prepared to cover a range of 1–90% chicken/beef by the same method. The lower concentration mixtures with 0.01%, 0.05%, 0.1%, 0.2%, 0.5%, 0.8% and 1.0% (*w/w*) for pork and chicken were prepared for determining the sensitivity of ddPCR assays. For evaluating the effect of thermal treatment on the accuracy of the quantitative method, binary meat mixtures with the mass proportion of 10% and 50% pork or chicken were steamed at 100 °C for 5, 10 and 20 min in a digital thermostatic water bath, respectively. Sixteen commercially available beef products purchased at retailers were used to verify the applicability of the established ddPCR method.

### 2.2. DNA Extraction

Total genome DNA was extracted from a sub-sample (100 mg) of each homogenized meat mixture using the DNeasy Tissue Kit (Qiagen, Hilden, Germany) following the manufactures protocol. The quality and quantity of the extracted DNA was subsequently determined by 1% agarose gel electrophoresis and a Nanadrop 2000 spectrophotometer (Thermo Fisher Scientific, Waltham, MA, USA). 

### 2.3. Specific Primers and Probes Synthesis

The single-copy nuclear genes of bovine *ACTB* (β-actin) gene (GenBank accession number: EH170825), porcine *ACTB* gene (GenBank accession number: DQ452569) and chicken *TGFB3* (transforming growth factor β3) gene (GenBank accession number: AY685072) were selected as the respective target sequences as described in previous research [33,34]. Primers and probes were designed using software Primer Premier 5.0 and synthesized by Sangon Biotech Co., Ltd. (Shanghai, China). The specificity of primers and probes was checked by BLAST searches against GenBank database and further validated by negative control in ddPCR assays. Probes were labelled with 5′-hexachlorofluorescein (HEX) or 6-carboxyfluorescein (FAM) as the reporter at the 5′-ends and black-hole-quencher 1 (BHQ1) as the quencher at the 3′-ends. All the primer and probe sequences are listed in Table 1.

### 2.4. ddPCR Assay

The ddPCR reactions were performed in a total volume of 20 μL, containing 10 μL 2 × ddPCR Master Mix (Bio-Rad, Hercules, CA, USA), 1.8 μL each primer (10 μM), 0.5 μL probe (10 μM), 1 μL template DNA (50 ng) and 4.9 μL ddH_2_O. A Bio-Rad QX200 ddPCR droplet generator (Bio-Rad, Hercules, CA, USA) was used to atomize the 20 μL mixture into approximately 20,000 droplets. After droplet generation, droplets were transferred to the 96-well PCR plate (Bio-Rad, Hercules, CA, USA). Conventional PCR were performed in a T100^TM^ Thermal Cycler (Bio-Rad, Hercules, CA, USA) according to the following conditions: a predenaturation at 95 °C for 10 min followed by 39 cycles of denaturation at 94 °C for 30 s and annealing/extension at 60 °C for 60 s, and a final enzyme inactivation step at 98 °C for 10 min. The amplification signals were read using a QX200 droplet reader (Bio-Rad, Hercules, CA, USA) and analyzed using its associated software QuantaSoftV 1.7.4.0971. The absolute copy numbers per reaction were estimated according to Possion statistics using the formula CPD = −ln(1-P/N), where the CPD was the copy number per droplet, P was the number of positive droplets and N was the total number of accepted droplets [35].

### 2.5. Establishment of Quantitative Formula

The ddPCR measures DNA of target meat species as absolute copy numbers. However, there exist a discrepancy between the DNA copies and the meat mass proportions. To avoid this bias, a constant (transfer coefficient) was introduced to directly transform the DNA copy numbers to the mass proportion of meats as described previously [21]. Briefly, in the meat mixtures of pork (or chicken) and beef, the mass ratio of pork (or chicken) and beef can be calculated by the following formula:

$$\frac{M_{T}}{M_{B}}=\frac{\frac{Q_{T}}{C_{T}}}{\frac{Q_{B}}{C_{B}}}=\frac{C_{B}}{C_{T}}\times \frac{Q_{T}}{Q_{B}},$$

where M_T_ and M_B_ refer to the mass of the tested meat species (pork or chicken) and beef, respectively; Q_T_ and Q_B_ refer to the copy number of the target gene (porcine *ACTB* or chicken *TGFB3* and bovine *ACTB*) detected in the ddPCR assays, respectively; C_T_ and C_B_ refer to the copy number of the target gene per unit mass of the corresponding meat species, respectively. For a specific type of meat species, the copy number of the target gene (single-copy gene) per unit mass could be considered invariable. Hence, under a fixed experimental procedure, the C_B_/C_T_ would be a constant, designated as transfer coefficient (K). Therefore, the final formula to determine mass of pork (or chicken) deviate in beef based on DNA copy number is:

$$\frac{M_{T}}{M_{B}}=K\times \frac{Q_{T}}{Q_{B}}.$$

To determine the K value for quantifying the pork or chicken in beef, ddPCR assays were conducted using a series of pork or chicken meat mixtures with concentration of 10%, 30%, 50%, 70% and 90% (*w/w*). To verify the accuracy and stability of the calculated K value, ddPCR assays were subsequently conducted using mixed samples containing pork or chicken powers with known mass composition (20%, 40%, 60% and 80%). Each sample was tested in six replicates.

### 2.6. Sensitivity, Repeatability and Reproducibility Analysis

To test the sensitivity of the ddPCR system, three indices including linearity range of quantification, limit of quantification (LOQ) and limit of detection (LOD) were determined. The linearity range of quantification was determined using a series of mixed meat samples with the pork or chicken content of 1%, 5%, 10%, 20%, 30%, 40%, 50%, 60%, 70%, 80% and 90% (*w/w*). Each sample was tested in triplicates and standard curves for tested meats were established by plotting the actual of mass fractions and the measured values of the meat fraction based on ddPCR assays. The LOQ was determined by evaluating the relative standard deviation (RSD) and the bias of mixed meat samples with the mass proportion of pork or chicken ranging from 0.01% to 10%. The LOD was defined as the lowest mass proportion that could be stably detected in all ddPCR assays. The repeatability and reproducibility of ddPCR system was assessed using meat mixtures ranging from 10 to 80% within the dynamic range. Each sample was tested in triplicates and independent experiments on two consecutive days by different operators were conducted.

### 2.7. Testing of Commercial Samples

To test the applicability of the established ddPCR method, sixteen commercially available beef products purchased at retailers were analyzed by the ddPCR assays. For DNA extraction, 100 mg of each sample was taken. Each sample was performed in triplicates and the measured results were then compared with the declared ingredients on the labels.

## 3. Results and Discussion

### 3.1. Species-Specific Identification

The mitochondrial DNA (mtDNA) is a widely used DNA marker for detection of animal-derived materials in foodstuffs and feedstuffs [36,37,38]. However, the mtDNA-based detection is insufficient to accurately quantify the traces in meat mixtures due to the variable content of mitochondria in different animal species and tissues [26]. Single-copy nuclear genes were selected in the present study since their copy number is relatively stable and would not vary between different gender, ages and tissues.

**Figure 1:**
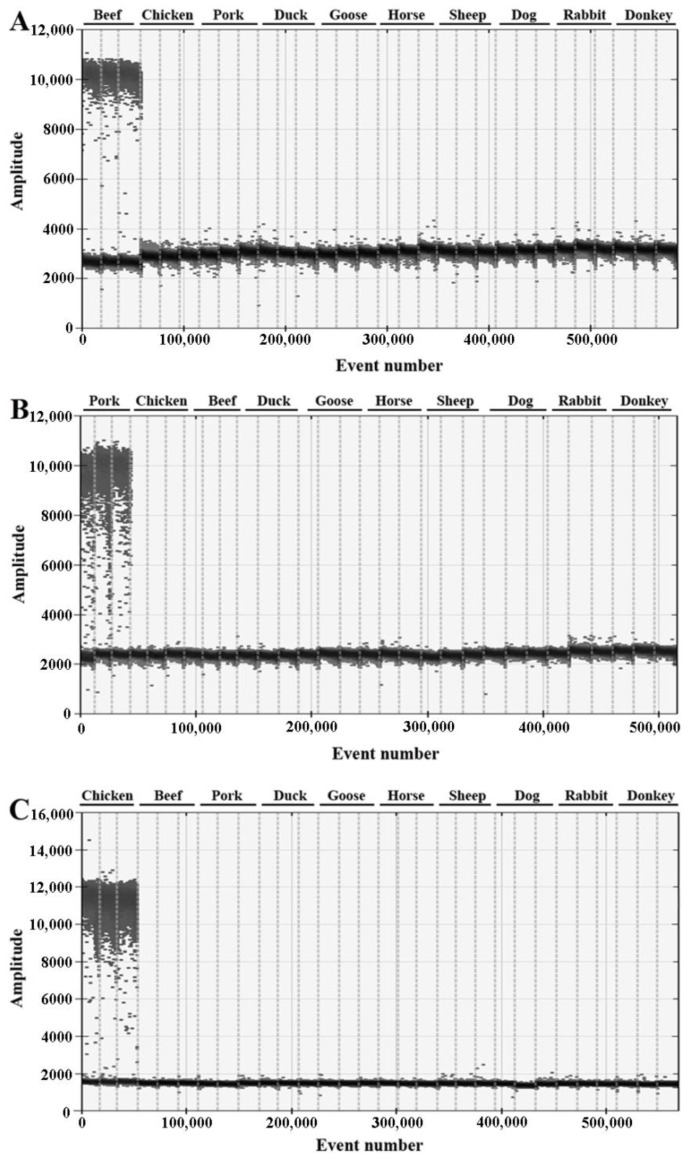
The specificity of beef (**A**), pork (**B**) and chicken (**C**) species-specific primers and probes in ddPCR assay. The non-targeting species were all with no amplification.

Specificity of the primers and probes targeted the porcine *ACTB*, chicken *TGFB3* and bovine *ACTB* were further confirmed using DNA from target and non-target meat samples including beef, pork, chicken, sheep, duck, goose, horse, dog, rabbit and donkey. As a result, species-specific primers and probes only amplified target sequences and showed no cross-reaction with any non-target species (Figure 1), indicating that the primer/probe sets could be used to specifically identify and quantify porcine- or chicken-derived materials in beef.

### 3.2. Determination and Verification of Transfer Coefficient (K)

In general, the meat adulteration quantification results are based on genome/genome equivalents. However, the genome/genome usually cannot represent the weight/weight directly because of differences in tissue composition and species genome size. In the present study, to directly transform the ratio of DNA copy numbers to the mass proportion of meats, a transfer coefficient (designed as K) was introduced as reported by Ren et al. [21].

To determine the K values, five mixtures of mass proportion (10%, 30%, 50%, 70% and 90%) of pork/beef and chicken/beef underwent ddPCR quantification of the content of corresponding targeted genes, respectively. As shown in Table 2, the average values of K_pork_ and K_chicken_ for six different meat fractions was 1.19 with the relative standard deviation (RSD) of 5.79% and 0.38 with the RSD of 6.32%, respectively. These results demonstrated that the K values for pork and chicken quantification in beef were stable regardless of mass ratio of pork/beef or chicken/beef.

To further verify the accuracy and stability of the K values, four mixed meat samples of known mass ratios (20%, 40%, 60% and 80%) were tested by the same ddPCR assays described above. As shown in Table 3, the final quantitative results for mixed pork–beef or chicken–beef samples based on the calculated K_pork_ and K_chicken_, respectively, were similar to the true mass proportion of the meats. The absolute value of deviation of pork was varied from 0.48% to 3.98% with an average of 2.48%, while that for chicken ranged from 0.13% to 6.99% with a mean of 2.76%. These results highly suggested that the values of K_pork_ and K_chicken_ were accurate and stable, which could be used for quantification of porcine and chicken derivatives in beef.

Therefore, two quantitative calculation formulae (1) M_pork_/M_beef_ = 1.19 × (Q_pork_/Q_beef_) and (2) M_chicken_/M_beef_ = 0.38 × (Q_chicken_/Q_beef_) were established for quantifying the content of pork and chicken in beef, respectively. Based on these formulae, the DNA copy numbers measured by ddPCR in the present study could be directly transferred to the mass proportion of meats unlike to previous reported ddPCR methods which needed to establish two standard curves for converting genome/genome to weight/weight [27,28,29].

### 3.3. Linearity Range, LOD, LOQ of Quantification

**Figure 2:**
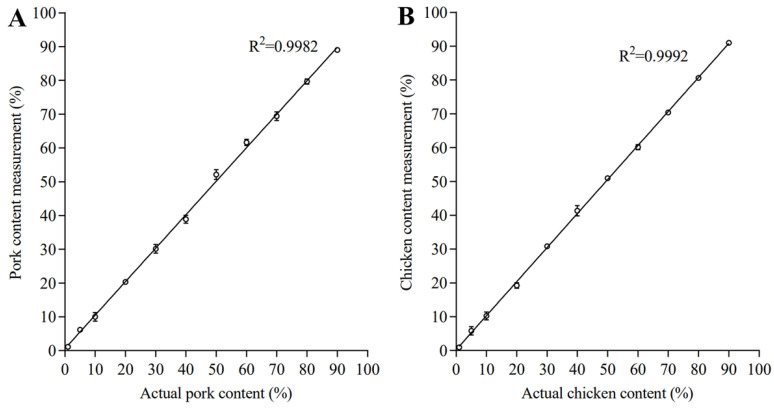
The linearity range of the ddPCR assays for quantification of pork (**A**) or chicken (**B**) fraction (*w/w*) in beef. Three replicates for each mass proportion were analyzed.

The linearity range of the ddPCR assays refers to the meat content intervals over which the method provides results with acceptable reliability [39]. As illustrated in Figure 2, the linear regression was established within the content of 1–90% both for pork and chicken, with the correlation coefficient (R^2^) of 0.9982 for pork and 0.9992 for chicken, respectively. It was thus clear that the ddPCR method established in this study displayed a wide linear region and hence suitable for quantification of porcine and chicken derivatives in beef.

The LOD was defined as the lowest percentage of pork or chicken in beef at which all sample replicates gave a positive qualitative result. As shown in Table 4, the ddPCR assays showed high sensitivity and the positive signal could be stably detected even in the level of 0.1% for both pork and chicken. Hence, the LODs of this ddPCR method were determined to be as low as 0.1% (*w/w*) for pork and chicken in beef.

According to the FAO Guideline on the performance criteria and validation of methods for the detection, identification and quantification of specific DNA sequence and specific proteins in foods [40], the LOQ of the ddPCR assays was defined as the lowest mass proportion of pork or chicken in beef that could be stably and reliably detected with an RSD ≤ 25%. Based on the calculated results, the values of LOQ for pork and chicken in beef both were 1% (*w/w*) which was an order of magnitude higher than the LODs for this ddPCR method (Table 4). These results were similar to the findings obtained in a recent study on the ddPCR quantification of meat species using a one-step conversion procedure to transform DNA copy number ratios into meat mass proportion, where the LOQs were 0.5% for chicken and 1% (*w/w*) for pork [32]. However, the LOQs detected in the present study were relatively lower than those detected in a previous ddPCR-based quantification study adopting a two-step conversion strategy in which the LOQ was 10% (*w/w*) for pork with the deviation of 17% [33]. In the present study, the bias of 10% (*w/w*) for pork was determined to be as low as 3.17%. It is thus obvious that ddPCR quantification methods with one conversion step can be more accurate and stable in the quantification of meat adulteration.

### 3.4. Repeatability and Reproducibility of Quantification

**Figure 3:**
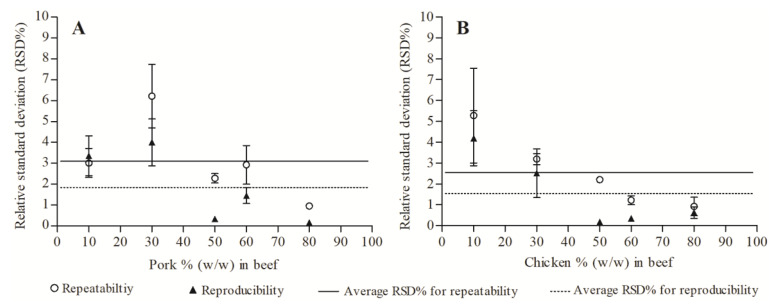
Repeatability and reproducibility of the ddPCR assay for pork (**A**) or chicken (**B**) fraction in beef.

In order to test the repeatability and reproducibility of the established ddPCR method, a series of meat fractions (10%, 30%, 50%, 60% and 80%) with defined content of pork and chicken were analyzed under repeatability conditions by two different experienced operators on two different days. As shown in Figure 3, the values of RSD calculated for repeatability among ddPCR replicates and for reproducibility among independent experiments for each meat fraction were all met well within the acceptance criterion of ≤25%. The RSDs in 50%, 60% and 80% pork mixtures were less than 3%, and those from the 60% and 80% chicken mixtures were below 2% for both repeatability and reproducibility. The relatively higher RSD values were observed in meat mixtures with the content of 30% pork and 10% chicken. Overall, our results demonstrated acceptable repeatability and reproducibility for the established ddPCR method.

### 3.5. Effect of Thermal Treatment on Quantification

In the meat industry, thermal treatment such as cooking and roasting is widely used to improve meat quality and safety. Generally, genomic DNA is easily degraded during the thermal processing of meat products, resulting in a loss of accuracy of quantification results [24,41]. To investigate the effect of thermal treatment on the accuracy of the established ddPCR method, binary meat mixtures with two defined mass proportions (10% and 50%) steamed at 100 °C for 5, 10 and 20 min were prepared for ddPCR assays. As shown in Table 5, the RSD values were all less than 25% in the measurement of 10% and 50% pork or chicken fraction mixtures subjected to thermal treatment for different times, indicating that a high level of accuracy in the quantification of pork and chicken in beef was maintained under thermal conditions. However, the RSD values were observed to be slightly higher along with increase in the treatment time, in agreement with the findings reported by Ren et al. [21].

### 3.6. Test Commercial Products

To test the applicability of the method in real products, sixteen commercial beef products were collected and analyzed by the ddPCR assays to determine the proportion of pork or chicken. The identification and quantification results are illustrated in Table 6. Out of sixteen beef products, one sample was incorrectly labeled with the pork proportion and one sample was adulterated with chicken. Based on the aforementioned quantitative formulae, the beef ball contained pork with the mass proportion of 31.67% and the minced beef contained chicken with the content of 65.90% while claiming to contain above 80% and 100% beef on the labels, respectively. These results confirmed the applicability and practicality of the established ddPCR method.

## 4. Conclusions

In this study, we developed an accurate and reliable ddPCR-based method for identifying and quantifying porcine and chicken derivatives in beef. The strategy was to introduce a fixed constant (transfer coefficient) to transform the ratio of DNA copy number (genome/genome) to the mass proportion (weight/weight) of targeted meat. Consequently, two quantitative formulae (1) M_pork_/M_beef_ = 1.19 × (Q_pork_/Q_beef_) and (2) M_chicken_/M_beef_ = 0.38 × (Q_chicken_/Q_beef_) were established for quantifying the content of pork and chicken in beef, respectively. The established method exhibited dynamic ranges from 1% (*w/w*) to 90% (*w/w*) with LOD of 0.1% (*w/w*) and LOQ of for 1% (*w/w*) for both pork and chicken in beef. A high level of accuracy of quantification was maintained under thermal conditions. Experiments on commercially available beef products confirmed the applicability of this ddPCR method. Overall, this method has great potential for application in routine assays for identification and quantification of beef adulteration.

## Figures and Tables

**Table 1:** Primer and probe sequences for the ddPCR assay.

Primer/Probe	Sequence (5′-3′)	Target Gene	Length
Beef-F	GTAGGTGCACAGTACGTTCTGAAG	*ACTB* [34]	96 bp
Beef-R	GGCCAGACTGGGCACATG		
Beef-P	HEX-CGGCACACTCGGCTGTGTTCCTTGC-BHQ1		
Pork F	GGAGTGTGTATCCCGTAGGTG	*ACTB* [34]	103 bp
Pork R	CTGGGGACATGCAGAGAGTG		
Pork-P	FAM-TCTGACGTGACTCCCCGACCTGG-BHQ1		
Chicken-F	GGCTGCAAGTCACCGTGGTA	*TGFB3* [33]	129 bp
Chicken-F	CCGCTAGCCAGAAGCTCAGC		
Chicken-P	FAM-CAGGAGCCACGTGAGCAGCACAG-BHQ1		

**Table 2:** The ratio of DNA copy numbers of unit mass with different proportions of pork and chicken, respectively.

Mass Proportion	Six Parallel Tests of Pork or Chicken (Copies/μL)						Six Parallel Tests of Beef (copies/μL)						K Value	K Mean (RSD)
Pork														
10%	29.7	31	30.3	31	32.2	31.7	332	350	345	344	355	361	1.25	1.19 (5.79%)
30%	80.3	71.1	76.7	77.8	81.2	87	201	190	189	205	223	233	1.12	
50%	109	121	123	127	130	140	122	130	136	143	141	158	1.11	
70%	120	120	121	122	123	134	62.8	64.5	64.6	65.3	64.2	71.1	1.24	
90%	137	137	140	142	143	164	18.2	18.5	19.4	19.7	20.3	22	1.23	
Chicken														
10%	230	247	255	225	221	226	756	792	711	736	746	831	0.36	0.38 (6.32%)
30%	281	282	272	286	221	247	266	209	170	265	274	258	0.39	
50%	375	392	377	401	384	394	130.1	146	121	144	142	129	0.35	
70%	535	582	583	593	601	600	100	106	102	105	105	99	0.41	
90%	581	591	596	632	638	591	23.8	22.5	24.8	24.3	29	28	0.38	

**Table 3:** Quantification results of mixed samples with known mass proportions of pork or chicken based on calculated K values.

Actual Value	Pork		Chicken	
	Measured Value (%)	Deviation	Measured Value (%)	Deviation
20%	20.44 ± 0.30	2.18%	18.60 ± 0.38	−6.99%
40%	38.68 ± 0.49	−3.29%	41.49 ± 0.64	3.73%
60%	62.39 ± 0.28	3.98%	60.08 ± 0.51	0.13%
80%	79.62 ± 0.48	−0.48%	80.15 ± 0.53	−0.19%

**Table 4:** The limit of detection (LOD) and limit of quantification (LOQ) of the ddPCR assay for pork or chicken fraction in beef.

Actual Value	Pork			Chicken		
	Measured Value (%)	Deviation	Detection Rate	Measured Value (%)	Deviation	Detection Rate
0.01%	0	100%	0%	0	−100%	0%
0.05%	0.01 ± 0.01	80.89%	33.3%	0	−100%	0%
0.1%	0.22 ± 0.21	115.72%	100%	0.17 ± 0.07	−70.88%	100%
0.2%	0.32 ± 0.02	58.28 %	100%	0.32 ± 0.13	−57.89%	100%
0.5%	0.68 ± 0.13	36.12%	100%	0.33 ± 0.13	33.99%	100%
0.8%	0.58 ± 0.12	−26.96%	100%	0.59 ± 0.15	−25.89%	100%
1%	1.13 ± 0.02	12.68 %	100%	0.98 ± 0.04	−2.39 %	100%
5%	5.66 ± 0.33	13.26%	100%	4.57 ± 0.16	−8.52%	100%
10%	9.68 ± 0.06	−3.17%	100%	10.91 ± 0.01	9.13%	100%

**Table 5:** Effect of thermal treatment on the accuracy of ddPCR-based quantification of pork or chicken fraction in beef.

Actual Value	Treatment Time (min)	Pork		Chicken	
		Measured Value (%)	Deviation	Measured Value (%)	Deviation
10%	5	9.50 ± 0.36	−4.97%	9.22 ± 0.31	−7.77%
	10	8.96 ± 0.33	−10.36%	9.00 ± 0.40	−10.03%
	20	8.39 ± 0.10	−16.10%	8.49 ± 0.04	−15.10%
50%	5	51.63 ± 0.62	3.26%	51.87 ± 0.48	3.75%
	10	53.48 ± 0.77	6.97%	52.79 ± 0.99	5.59%
	20	53.99 ± 0.17	7.99%	42.77 ± 5.26	−14.46%

**Table 6:** Quantification results of commercial beef products.

Sample	Pork % (*w/w*)		Chicken % (*w/w*)	
	Declared Value	Measured Value	Declared Value	Measured Value
Beef roll	0	0	0	0
Beef ball_1	0	0	0	0
Beef ball_2	19.00%	31.67%	0	0
Beef kebabs_1	0	0	0	0
Beef kebabs_2	0	0	0	0
Beefsteak	0	0	0	0
Minced beef	0	0	0	65.90
Beef stick	0	0	0	0
Beef slice	0	0	0	0
Veal sausage	0	0	0	0
Beef sausage	0	0	49.40%	50.25%
Canned beef	0	0	0	0
Dried beef jerky	LC	17.47%	0	0
Dried beef floss	0	0	0	0
Cured beef	0	0	0	0
Bresaola	0	0	0	0

LC: Lacking defined content on the label.

## Data Availability

Data is contained within the article.
